# Fossil and non-fossil sources of the carbonaceous component of PM_2.5_ in forest and urban areas

**DOI:** 10.1038/s41598-023-32721-2

**Published:** 2023-04-04

**Authors:** Ji-Yeon Cha, Kyuyeon Lee, Seung-Cheol Lee, Eun-Ju Lee, Kwang-Jin Yim, Ilhan Ryoo, Minhye Kim, Jinho Ahn, Seung-Muk Yi, Chan-Ryul Park, Neung-Hwan Oh

**Affiliations:** 1 Department of Environmental Planning, Graduate School of Environmental Studies, Seoul National University, Seoul, 08826 Republic of Korea; 2 Environmental Planning Institute, Seoul National University, Seoul, 08826 Republic of Korea; 3 School of Earth and Environmental Sciences, Seoul National University, Seoul, 08826 Republic of Korea; 4 Department of Environmental Health, Graduate School of Public Health, Seoul National University, Seoul, 08826 Republic of Korea; 5 Institute of Health and Environment, Seoul National University, Seoul, 08826 Republic of Korea; 6 Urban Forests Division, National Institute of Forest Science, Seoul, 02455 Republic of Korea

**Keywords:** Biogeochemistry, Environmental sciences

## Abstract

Atmospheric particulate matter (PM_2.5_) can damage human health. Biogenic organic compounds emitted from trees may increase the concentration of PM_2.5_ via formation of secondary aerosols. Therefore, the role of biogenic emissions in PM_2.5_ formation and the sources of PM_2.5_ need to be investigated. Dual carbon isotope and levoglucosan analyses are powerful tools to track the sources of total carbon (TC) in PM_2.5_. We collected a total of 47 PM_2.5_ samples from 2019 to 2020 inside a pine forest and in urban areas in South Korea. The average δ^13^C and Δ^14^C of TC in PM_2.5_ at the Taehwa Research Forest (TRF) were − 25.7 and − 380.7‰, respectively, which were not significantly different from those collected at Seoul National University (SNU) in urban areas. Contribution of fossil fuel, C_3_-, and C_4_- plants to carbonaceous component of PM_2.5_ were 52, 27, and 21% at SNU, whereas those were 46, 35, and 19% at TRF, respectively. The biomass burning tracer, levoglucosan, was most abundant in winter and correlated with the contribution of C_4_ plants derived carbon. Results indicate that biogenic aerosols emitted from trees is less likely to be an important source of PM_2.5_ and that trees can act as a bio-filter to reduce PM_2.5_.

## Introduction

Pollution due to fine particulate matter with a diameter of 2.5 μm or less (PM_2.5_) is a significant challenge, which affects human health and ecosystem, visibility, and climate change^1,2^. The mean annual concentrations of PM_2.5_ ranged from 30.6 to 37.2 μg m^−3^ between 2013 and 2017 in Seoul, the capital of South Korea^3^, which still exceed the regulatory standard of 15 μg m^−3^ specified by the Korean government. Higher concentrations of PM_2.5_ are frequently observed^4,5^.

Trees can remove atmospheric PM_2.5_ by directly adsorbing PM_2.5_ on the surface of leaves and branches, and by absorbing some of the PM_2.5_ through the stomata^6,7^. The mean annual PM_2.5_ levels removed by trees in the urban areas are estimated at 0.27 and 0.15 g m^−2^ in the conterminous United States and in 86 Canadian cities, respectively^6,8^. In addition, plants alter the local microclimate conditions by reducing air temperature and increasing relative humidity via canopy transpiration, thus increasing the deposition of PM onto leaves^9^. Therefore, this process can reduce the concentration of PM_2.5_ in urban areas^6,9,10^. For example, in central Sydney, Australia, the PM_2.5_ concentrations were lower in urban areas with a relatively higher density of green space^10^. Therefore, urban forest expansion has been regarded as one of the government policies to reduce PM_2.5_ concentrations in many countries^11^. South Korea is no exception, and the Korea Forest Service has proposed an increase in urban green space to 15 m^2^ per capita from the current 9.9 m^2^ per capita by 2027^12^.

However, trees indirectly increase PM_2.5_ concentrations. Trees emit biogenic volatile organic compounds (BVOCs) such as isoprene and terpenes as well as alcohols, carbonyls, and acids^13^. The oxidation products of these BVOCs can form and grow biogenic secondary organic aerosols (SOAs)^14^. SOAs constitute up to 85% of organic carbon and ~ 35% of PM_2.5_^15^. SOAs may contribute to the formation of carbonaceous materials in fine particles in the presence of elevated levels of anthropogenic emissions of NOx and oxidants (OH radicals and ozone). For example, the formation of biogenic SOAs can be enhanced by 60–200% due to the emission of NOx and oxidants^16^. The mass concentration of submicron particles is also increased by 25–200% at polluted sites downwind of Manaus, Brazil^17^. Furthermore, studies using radiocarbon (^14^C) reported that a high biogenic fraction can contribute to the formation of carbonaceous component of PM_2.5_ in cities, accounting for up to 80% of the aerosol carbon^18–20^.

These findings bring a question on how much biogenic emissions contribute to the formation of PM_2.5_ in the atmosphere and whether the sources of PM_2.5_ in forests are different from urban areas. ^14^C is a powerful tracer of the carbon cycle, which separates fossil-fuel-derived carbon from recently photosynthesized carbon. However, the ^14^C analysis cannot distinguish if the sources of carbon in PM_2.5_ are generated from biogenic emissions or biomass burning because both are derived from recently photosynthesized carbon^21,22^. Thus, a biomarker is needed to further identify the sources of biogenic PM_2.5_. Levoglucosan has been widely used to trace the pyrolytic emissions of PM_2.5_ released by biomass burning^23–26^. In addition to radiocarbon and levoglucosan analyses, the stable carbon isotope ratio (δ^13^C) can provide further information on the sources of carbon, *i.e.*, C_3_ and C_4_ plants, because of their distinct isotopic fractionation^27,28^. C_3_ plants (*e.g.*, most trees) incorporate CO_2_ into the three-carbon compound, 3-phosphoglyceric acid, during the first stage of photosynthesis, whereas C_4_ plants (*e.g.*, maize and sorghum) initially fix CO_2_ in the mesophyll cell as a four-carbon compound, oxaloacetate, which results in distinctive δ^13^C^29,30^.

The objectives of this study are (1) to investigate the temporal variation in the concentrations of total carbon (TC) including organic and elemental carbon in PM_2.5_, (2) to compare carbon isotope ratios (δ^13^C and Δ^14^C) between a pine forest and urban area, and (3) to track the sources of TC in PM_2.5_ by analyzing δ^13^C, Δ^14^C, and levoglucosan concentrations.

## Methods and materials

### Sampling sites and collection of PM_2.5_

**Figure 1:**
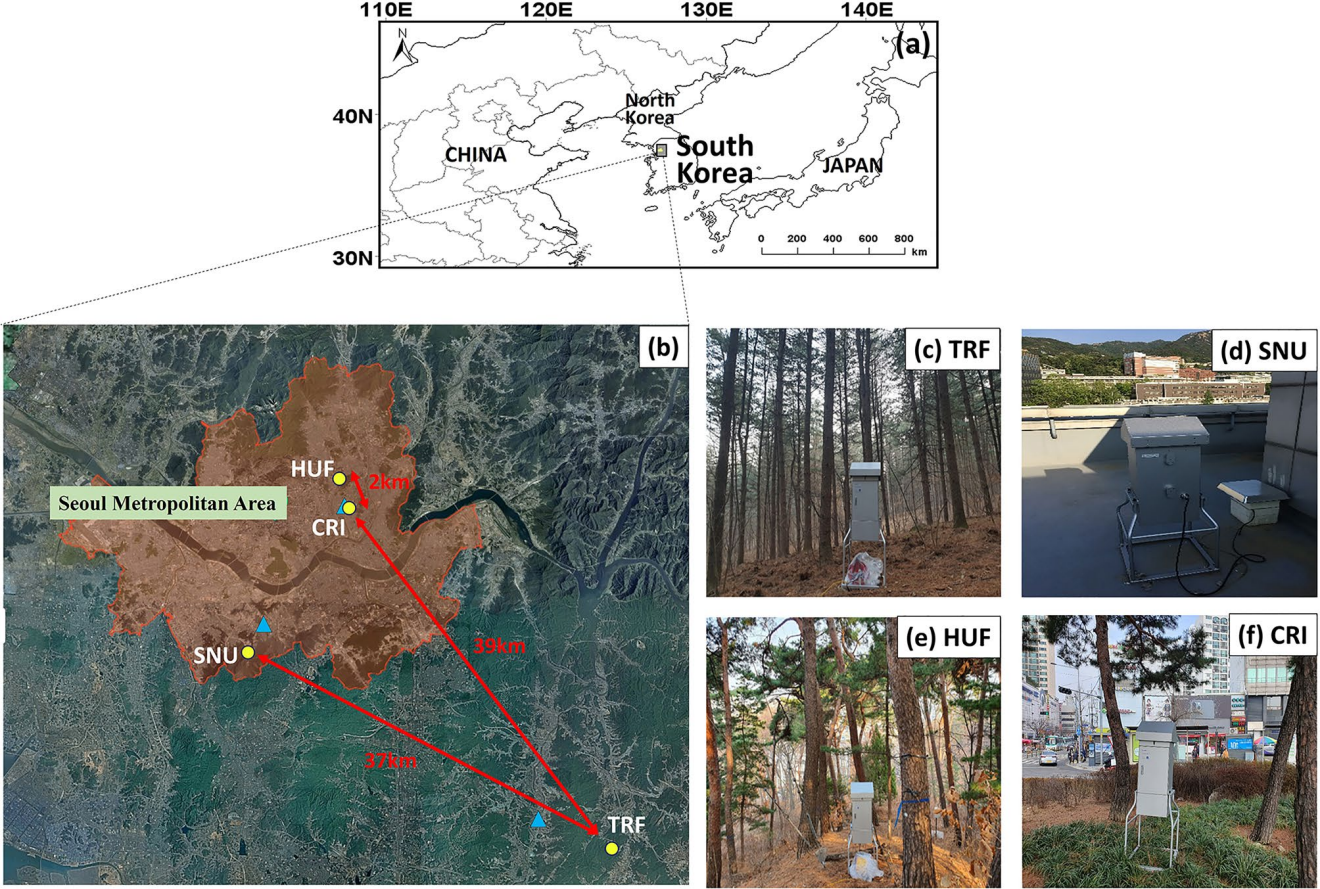
(**a**) The location of study sites in South Korea, (**b**) PM_2.5_ sampling points for high-volume air sampler (yellow circles) and the national PM_2.5_ monitoring sites (blue triangles). The high-volume air sampler was set at (**c**) the Taehwa Research Forest (TRF), (d**)** the Seoul National University (SNU), (**e**) the Hongneung urban forest (HUF) and (f) Cheongnyangni traffic island (CRI). The map of East Asia in (**a**) was created by MeteoInfo 3.5.5 (http://www.meteothink.org) and the satellite imagery in (**b**) was created on the Vworld platform (http://map.vworld.kr) provided by the Korean Ministry of Land, Infrastructure and Transport.

Samples were collected from four sites (Fig. 1a,b) as follows:

1. A Korean pine (*Pinus koraiensis*) forest located at the Taehwa Research Forest (TRF) (37°30.5ʹN, 127°31.6ʹE, Fig. 1c), where pines were planted in 1960s^31^. TRF is a part of Seoul National University Forests, and is located in the suburban area^32^.
2. The rooftop of a building located in the Seoul National University (SNU) campus (37°45.8ʹN, 126°95.0ʹE, Fig. 1d) in the southern part of Seoul (~ 10 million population), the capital of South Korea.
3. A pine forest at the Hongneung Urban Forest (HUF) near Mt. Cheonjang (37°59.6ʹN, 127°04.5ʹE, Fig. 1e) in Seoul, which is a fragmented forest surrounded by urban residential area. Dominant species include *Pinus densiflora* and *P. koraiensis*.
4. A green space with pine trees located at Cheongnyangni Traffic Island (CRI) (37°58.0ʹN, 127°04.5ʹE, Fig. 1f) in Seoul, which is a small triangle area (0.06 ha) between roads.

According to the Food and Agriculture Organization of the United Nations^33^, forest is defined as “land spanning more than 0.5 hectares with trees higher than 5 m and a canopy cover of more than 10 percent, or trees able to reach these thresholds in situ”. In that sense, TRF is a pine forest in rural area. HUF is also a pine forest, but located in urban area. CRI is not a forest but a simple green space with pine trees near busy traffic roads in urban area. SNU is in the urban area. The population size and density are often used to define a place as ‘urban’. Seoul is one of the largest metropolitan areas (~ 10 million population) in the world.

A high-volume air sampler (HVAS; HV-1700RW, Sibata, Tokyo, Japan) was used to collect the PM_2.5_ on quartz filters at a flow rate of 1000 L min^−1^^23,28,34,35^. An HVAS was placed on the rooftop of a building at SNU, and the other HVAS was installed inside the forests or green space. The high-volume air samplers were automatically calibrated by a built-in flowmeter, temperature sensor, and atmospheric pressure sensor. No plant parts were sampled in this study. Between 2019 and 2020, a total of 47 samples were collected with a time resolution of 24–72 h (usually 24 h). To collect PM_2.5_ in the atmosphere, the quartz filters were pre-baked at 400 °C for 4 h prior to use for removing the organic contaminants. Quartz filters are widely used to measure carbonaceous aerosols^23,28,34,35^ because their composition is carbon-free, and can remain stable at temperatures up to 900 °C for carbon isotope analysis. However, the quartz filters were not used to measure the mass of PM_2.5_ because the filter size is too large (203 mm by 254 mm) to be precisely weighed on an analytical balance. Instead, beta attenuation mass monitors (BAM) are widely used to measure the concentration of PM_2.5_ indirectly. Both methods complement each other, BAM for relatively simple estimation of PM_2.5_ concentration and HVAS for isotopic analysis. Similar patterns were observed between PM_2.5_ by BAM and TC in PM_2.5_ by HVAS (Fig. 3a–c), but they were not used for cross-calibration.

Most of the samples were collected at TRF (n = 21) and SNU (n = 18) to compare the sources of carbonaceous component of PM_2.5_ between forest and urban areas (Table 1). A total of 11 samples were simultaneously collected at both TRF and SNU. The additional samples were collected inside the green space or forests at CRI (n = 6) and HUF (n = 2), respectively, in the urban areas during fall and winter (Table 1). After sampling, filters were wrapped in pre-baked aluminum foil and stored at − 20 °C. Field blank filters were also prepared by placing the filter in the sampler for 24 to 72 h without gas flow, and then stored as described above. The mass of carbon on the field blank filters was negligible compared with that of collected PM_2.5._

**Table 1:** Concentrations and key properties of PM_2.5_ collected by high volume air samplers at TRF, HUF, CRI, and SNU.

Date (mm/dd/yyyy)	Collecting period (hours)	TC in PM_2.5_^†^ (%)	TC (μg C m^−3^)	δ^13^C (‰)	Δ^14^C (‰)	Levoglucosan (ng m^−3^)	Season
Taehwa Research Forest (TRF), Gwangju-si (n = 21)							
08/02/2019	24	32	4.5	− 26.6	− 489.9	NA^‡^	Summer
08/05/2019	24	19	5.3	− 26.7	− 250.3	6.41	Summer
01/24/2020	24	13	7.0	− 25.6	− 323.0	NA	Winter
02/07/2020	24	16	5.3	− 24.5	− 382.9	66.52	Winter
02/13/2020	23	10	5.3	− 26.0	− 441.2	44.94	Winter
02/20/2020	24	16	8.7	− 26.1	− 360.2	62.92	Winter
02/21/2020	24	11	6.4	− 25.5	− 349.6	NA	Winter
02/22/2020	48	9	3.5	− 25.0	− 357.3	60.02	Winter
04/10/2020	24	24	6.6	− 23.2	− 227.8	60.47	Spring
04/13/2020	24	45	4.9	− 25.0	− 204.5	39.96	Spring
05/04/2020	24	35	5.6	− 25.5	− 273.9	NA	Spring
05/21/2020	24	42	3.8	− 26.1	− 381.8	18.94	Spring
07/08/2020	24	22	3.9	− 26.3	− 536.2	9.57	Summer
08/18/2020	24	15	3.4	− 26.6	− 576.4	8.95	Summer
09/14/2020	24	29	3.2	− 25.7	− 514.0	13.98	Fall
10/13/2020	24	34	4.1	− 26.0	− 373.2	37.92	Fall
10/14/2020	24	29	4.3	− 26.0	− 311.4	NA	Fall
10/15/2020	24	29	4.1	− 26.6	− 360.3	NA	Fall
10/16/2020	24	26	4.7	− 26.8	− 410.3	51.68	Fall
11/14/2020	48	7	3.0	− 26.4	− 375.0	34.74	Fall
12/11/2020	24	10	7.3	− 24.6	− 494.9	53.08	Winter
Mean		22	5.0^b^	− 25.7	− 380.7^b^	38.01	
Hongneung Urban Forest (HUF), Seoul (n = 2)							
02/20/2020	24	20	8.6	− 25.7	− 417.2	65.82	Winter
10/13/2020	24	49	5.4	− 25.7	− 370.0	40.00	Fall
Mean		34	7.0^ab^	− 25.7	− 393.6^ab^	52.91	
Cheongnyangni Traffic Island (CRI), Seoul (n = 6)							
02/21/2020	24	17	8.2	− 24.8	− 433.8	NA	Winter
02/22/2020	48	16	5.8	− 25.1	− 426.4	38.46	Winter
02/24/2020	34	34	6.9	− 26.0	− 451.3	NA	Winter
10/14/2020	23	66	5.4	− 25.0	− 421.3	NA	Fall
10/15/2020	24	86	8.6	− 25.9	− 465.6	NA	Fall
10/16/2020	24	61	10.4	− 26.0	− 467.5	61.11	Fall
Mean		47	8.2^ab^	− 25.2	− 444.3^a^	49.78	
Seoul National University (SNU) campus, Seoul (n = 18)							
01/14/2019	24	19	25.6	− 23.7	589.7^††^	4.55	Winter
02/22/2019	24	39	23.9	− 23.6	− 313.7	75.91	Winter
03/05/2019	24	8	10.4	− 23.7	− 503.6	NA	Spring
04/22/2019	24	28	10.0	− 25.9	− 239.3	9.69	Spring
05/24/2019	24	29	14.7	− 26.0	− 430.1	NA	Spring
06/05/2019	24	27	9.2	− 25.8	− 561.9	NA	Summer
07/17/2019	24	14	7.4	− 25.5	− 370.6	NA	Summer
08/08/2019	24	14	5.5	− 26.1	− 459.4	5.57	Summer
02/07/2020	24	59	16.6	− 23.6	− 352.5	64.52	Winter
02/13/2020	24	12	7.0	− 25.2	− 370.1	NA	Winter
04/10/2020	24	58	16.4	− 22.2	− 284.9	66.24	Spring
04/13/2020	24	50	7.0	− 24.1	− 290.5	38.25	Spring
05/04/2020	24	69	11.0	− 25.3	− 400.0	NA	Spring
05/21/2020	24	50	5.5	− 26.2	− 415.6	7.41	Spring
07/08/2020	24	24	5.3	− 25.7	− 543.7	8.61	Summer
08/18/2020	24	12	2.8	− 27.3	− 635.9	4.38	Summer
09/14/2020	24	26	3.4	− 26.1	− 503.6	6.63	Fall
11/13/2020	70	12	5.6	− 25.3	− 481.6	18.50	Fall
12/11/2020	24	16	12.3	− 24.6	− 439.1	70.72	Winter
Mean		30	9.7^a^	− 25.1	− 422.0^ab^	36.36	

^‡^NA: Not assessed.

^†^Calculated by dividing the TC (μg C m^-3^) by the concentration of PM_2.5_ at the national monitoring sites in South Korea near TRF, HUF&CRI, and SNU.

^††^This extraordinary Δ^14^C sample was excluded from statistical analysis. The details are described in the Supplementary Information.

### PM_2.5_ monitoring data in South Korea

The AirKorea website (http://www.airkorea.or.kr) provides nationwide PM_2.5_ data collected from outdoor monitoring sites via β-ray absorption. The method is used to estimate the concentration of PM_2.5_ based on the relationship between attenuation of beta radiation and PM_2.5_ deposited on a glass fiber filter tape within an instrument. We downloaded the daily PM_2.5_ concentration data of the three national monitoring sites (Fig. 1b) from the AirKorea website. One of the monitoring sites for both HUF and CRI was located next to the road, and the monitoring sites for TRF and SNU, respectively, were located at the rooftop of each Community Center. The monitoring site for TRF was located outside the forest, 6.6 km apart from TRF in the rural area, whereas the other monitoring sites were located within 2.4 km of our sampling sites in the urban area. All three national monitoring sites are located outside forests, providing information on PM_2.5_ concentration.

Based on the daily PM_2.5_ data reported by the AirKorea, the sampling dates for HVAS were chosen when the concentration of PM_2.5_ was higher than ~ 15 μg m^−3^, which is the air quality guideline of PM_2.5_. The HVAS was also used in order to collect enough amount of carbon for isotopic analysis which was >  ~ 1 mg-C.

### Dual carbon isotope analysis

The PM_2.5_ samples were acidified with 10% HCl to remove inorganic carbon, and then dried at 50 °C^36–38^. Each dried filter was transferred to a pre-burned quartz tube containing CuO as an oxidant and silver wire. The quartz tube was evacuated, flame-sealed, and heated at 850 °C for 4 h to oxidize total carbon (TC) including organic and elemental carbon. The resulting CO_2_ in the quartz tube was sent to the national ocean sciences accelerator mass spectrometry facility (https://www2.whoi.edu/site/nosams/) to measure dual carbon isotope ratios (δ^13^C and Δ^14^C) of TC in PM_2.5_ via accelerator mass spectrometry. The IAEA-C8 oxalic acid was routinely used as a reference standard material, and its δ^13^C and ^14^C activity was measured within the recommended range (https://nucleus.iaea.org/sites/ReferenceMaterials/Pages/IAEA-C-8.aspx). To our knowledge, this is the first study reporting δ^13^C and Δ^14^C of PM_2.5_ over an entire year in South Korea.

### Source apportionment of TC in PM_2.5_ using IsoSource and Bayesian statistics

**Figure 2:**
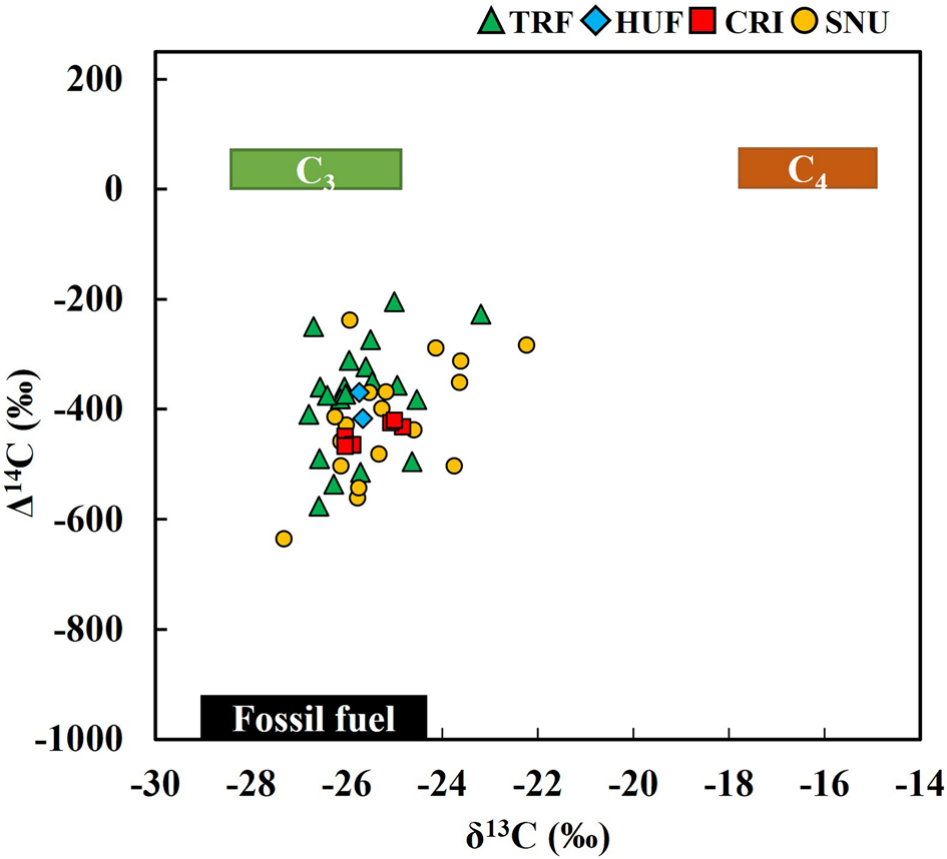
Isotopic source diagram for the PM_2.5_ samples. The black, green, and brown bars indicate three endmembers including fossil-, C_3_ plants-, and C_4_ plants-derived carbon, respectively.

First, the sources of TC in PM_2.5_ were separated into fossil fuel and non-fossil fuel sources based on radiocarbon results^38^. The contribution of each non-fossil fuel source (i.e., C_3_ and C_4_ plants) was further quantified by incorporating the results of stable carbon isotope analysis using IsoSource and Bayesian mixing models. Three endmembers were used: fossil fuel, C_3_ plants, and C_4_ plants (Fig. 2). The δ^13^C of the three endmembers was set to − 29.0 ± 1.3‰ for fossil fuel, − 26.7 ± 1.8‰ for C_3_ plants, and − 16.4 ± 1.4‰ for C_4_ plants^27,39^. The Δ^14^C of carbon derived from fossil fuel was set to − 1000‰, while the Δ^14^C values of C_3_ and C_4_ plants were set to 30‰^36^, similar to the Δ^14^C-CO_2_ in the troposphere because plants absorb atmospheric CO_2_ during photosynthesis. Both the δ^13^C and Δ^14^C results were incorporated to draw quantitative results of carbon sources. The dual isotopic mixing model is commonly used in ecological studies to determine the proportions of various sources in a mixture^28,39,40^.

IsoSource, a popular mixing model, was used to quantify TC in PM_2.5_ sources into fossil fuels, C_3_ plants, and C_4_ plants (https://www.epa.gov/eco-research/stable-isotope-mixing-models-estimating-source-proportions)^41^. All possible combinations of source proportions were calculated with IsoSource using a 0.1‰ tolerance. The radiocarbon results were used as additional constraints to further reduce the range of contributions from the fossil fuel sources. The IsoSource results were used as prior information via Bayesian inferences using Markov Chain Monte Carlo (MCMC) method (Figs. S1 and S2 in Supplementary Information). In this MCMC model, the sources were also separated into fossil fuels, C_3_ plants, and C_4_ plants. To estimate seasonal source contributions to TC in PM_2.5_, we combined all the data points from each season in the MCMC calculation. Annual source apportionment was conducted by combining all the data points at each site. MCMC was implemented using the *simmr*-0.4.5 (https://cran.r-project.org/web/packages/simmr/index.html) in R-4.1.1^42^.

### Levoglucosan analysis

The dual carbon isotope analysis does not distinguish if the sources of carbon in PM_2.5_ are generated from biogenic emissions or biomass burning. In order to investigate the possibility of the biomass burning as a source of carbon in PM_2.5_, levoglucosan analysis was employed to complement carbon isotope analysis^23–26^. The levoglucosan concentration, which is a biomarker of biomass burning, was analyzed^23–26^. The samples were sonicated with dichloromethane: methanol (3:1, v/v) at 20 °C for 1 h. The extract was concentrated using nitrogen gas in TurbovapII (Zymark Co., USA) and filtered using a 0.45-μm PTFE syringe filter (Pall Corporation, USA). After filtration, the extract was reconcentrated to a final volume of 1 mL using Turbovap II and Reacti-Therm (Thermo Scientific, TS-18822, USA) and stored in a freezer until analysis.

Derivatization via silylation was conducted to analyze polar compounds. A 50 μL aliquot of the final extract volume was completely dried by gently blowing nitrogen gas, followed by reaction with 50 μL of N, O-bis-(trimethylsilyl) trifluoroacetamide (BSTFA) combined with 1% trimethylchlorosilane (TMCS) (Sigma Aldrich, USA) and 50 μL of pyridine (HPLC grade, Sigma Aldrich, USA) at 75 ℃ for 90 min. After derivatization, the concentration of levoglucosan was determined using a GC/MS (7890B/5977B, Agilent, USA) operating at an ionization energy of 70 eV in EI mode.

### Statistical analysis

Differences in PM_2.5_ concentrations and carbon isotope signatures among sites and seasons were analyzed via one-way analysis of variance (ANOVA), followed by Tukey’s HSD (honest significant difference) test. Type II error (*i.e.*, false negative) can occur when the sample size is too small. The number of samples per season ranged from 2 to 7 in this study, making the statistical analysis on them prone to type II error. Although we fully recognize the limitations of the analysis due to the relatively small number of samples per season, we still have attempted to provide seasonal information of TC in PM_2.5_ as Korea is located in the Asian monsoon climate, where the highest PM_2.5_ concentrations were reported in winter and decreased to a minimum in summer^3^. A linear regression analysis was used to assess the relationship between levoglucosan concentration and fractional contribution of carbon derived from C_3_ or C_4_ plants to PM_2.5_. The analyses were conducted using R-4.1.1^42^.

## Results

### δ^13^C and Δ^14^C of TC in PM_2.5_

Both δ^13^C and Δ^14^C values of TRF were not significantly different from those of SNU on the same dates (n = 11) (Fig. 2 and Table 1). In contrast, the Δ^14^C values of CRI were significantly lower than those of TRF on the same collection dates in fall and winter (n = 5 each), with the mean Δ^14^C values of CRI and TRF at − 444.3‰ and − 380.7‰, respectively (Table 1). The δ^13^C and Δ^14^C values of TC in PM_2.5_ varied temporally (Table 1) Enriched δ^13^C and Δ^14^C values were observed in winter and spring, while, in summer, δ^13^C and Δ^14^C were significantly depleted than other seasons (Fig. S3 in Supplementary Information).

### Concentration of TC and PM_2.5_

A total of 47 PM_2.5_ samples were collected during the study period (Table 1). The mean concentrations of TC in PM_2.5_ were 5.0, 7.0, 8.2, and 9.7 μg C m^−3^ at TRF, HUF, CRI, and SNU, respectively (Table 1). The mean concentration of TC in PM_2.5_ at SNU was about two-fold higher than at TRF (*p* < 0.05) (Table 1). The seasonal variation of TC in PM_2.5_ was relatively small in summer (Jun.–Aug.) and larger in winter (Dec.–Feb.) (Fig. S4 in Supplementary Information) and the concentration of TC in PM_2.5_ in winter was approximately 1.5-fold higher than in summer (*p* < 0.05).

**Figure 3:**
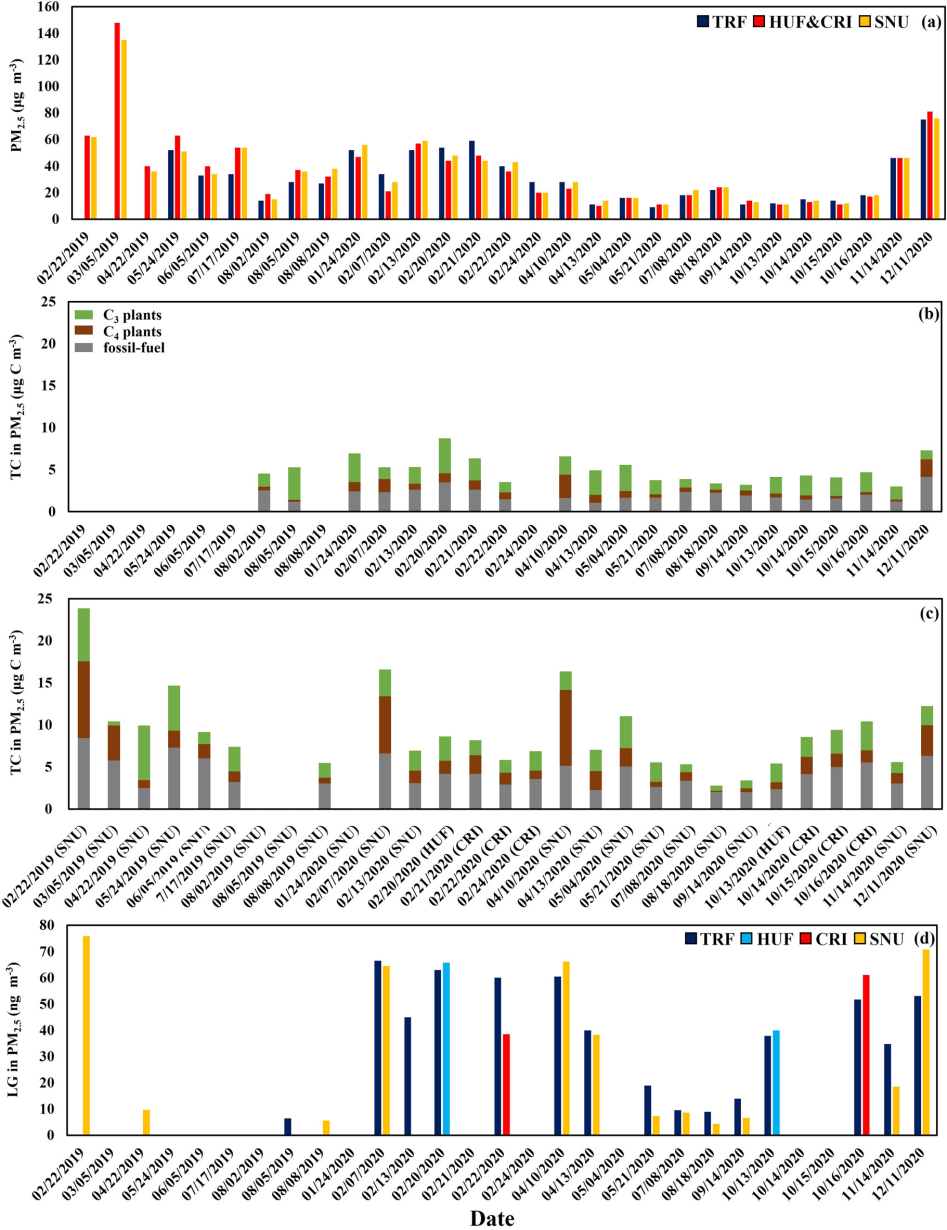
(**a**) The concentrations of PM_2.5_ (μg m^−3^) at the national PM_2.5_ monitoring sites near SNU, HUF&CRI (one site; section "PM_2.5_ monitoring data in South Korea"), and TRF. (**b**) The concentration (μg C m^−3^) of TC in PM_2.5_ at TRF. (**c**) The concentrations of TC in PM_2.5_ at SNU, HUF, and CRI. The green, brown, and grey bars in (**b**) and (**c**) represent carbon derived from C_3_ plants, C_4_ plants, and fossil fuel, respectively. (**d**) The concentration (ng m^−3^) of levoglucosan.

The mean annual concentrations of PM_2.5_ observed by the β-ray absorption method were 22, 20, 22 μg m^−3^, respectively, at the national PM_2.5_ monitoring sites located near TRF, HUF&CRI, and SNU in 2020, which were about 0.3–6.6 km away from our sampling sites (Fig. 3a). The concentration of PM_2.5_ was not significantly different among monitoring sites (*p* > 0.1) regardless of seasons (Fig. 3a). The contribution of TC to PM_2.5_ near monitoring sites ranged from 7 to 45% at TRF, from 16 to 86% at HUF and CRI, and from 8 to 69% at SNU (Table 1). The contribution of TC to PM_2.5_ was the highest in spring at TRF and SNU (Table 1; Fig. 3).

### Source apportionments

The contribution of the three sources (fossil fuel, C_4_ plants, and C_3_ plants) to TC in PM_2.5_ was calculated. The contribution of carbon derived from fossil fuel ranged from 21 to 73% (Fig. 3b,c), with an average of 46%, 46%, 51%, and 51% at TRF, HUF, CRI, and SNU, respectively, based on the MCMC-Bayesian model (Table 2). The relative contribution of carbon from fossil fuel to TC in PM_2.5_ was higher in summer than in winter at TRF and SNU (Table 2), which was expected from the depleted δ^13^C and Δ^14^C levels in summer (Fig. S3c in Supplementary Information).

**Table 2:** The means and the standard deviations of the concentration (μg C m^−3^) of each source. The mean relative contribution (%) of each source to carbonaceous component of PM_2.5_ is in parentheses.

	Fossil fuel	C_4_ plants	C_3_ plants
TRF			
Spring (Mar.–May)	1.5 ± 0.3 (29)	1.2 ± 0.9 (22)	2.5 ± 0.6 (49)
Summer (Jun.–Aug.)	2.1 ± 0.5 (52)	0.4 ± 0.1 (10)	1.8 ± 1.2 (38)
Fall (Sep.–Nov.)	1.7 ± 0.3 (44)	0.4 ± 0.1 (12)	1.9 ± 0.6 (44)
Winter (Dec.–Feb.)	2.7 ± 0.8 (44)	1.2 ± 0.4 (19)	2.3 ± 1.1 (37)
Total	2.1 ± 0.7 (46)	0.8 ± 0.6 (19)	2.1 ± 1.0 (35)
HUF			
Fall (10/13/2020)	2.4 (44)	0.8 (15)	2.2 (41)
Winter (02/20/2020)	4.2 (48)	1.6 (18)	2.9 (34)
Total	3.3 ± 0.9 (46)	1.2 ± 0.4 (17)	2.6 ± 0.3 (38)
CRI			
Fall (10/14–16/2020)	4.9 ± 0.6 (51)	1.7 ± 0.3 (18)	2.9 ± 0.4 (31)
Winter (02/21–24/2020)	3.6 ± 0.5 (51)	1.5 ± 0.5 (21)	1.9 ± 0.3 (28)
Total	4.2 ± 0.9 (51)	1.6 ± 0.4 (21)	2.4 ± 0.6 (29)
SNU			
Spring (Mar.–May)	4.4 ± 1.8 (43)	3.0 ± 2.7 (25)	3.3 ± 1.9 (32)
Summer (Jun.–Aug.)	3.5 ± 1.3 (62)	1.0 ± 0.5 (15)	1.5 ± 0.8 (24)
Fall (Sep.–Nov.)	2.5 ± 0.5 (56)	0.8 ± 0.4 (20)	1.1 ± 0.2 (24)
Winter (Dec.–Feb.)	6.1 ± 1.9 (43)	5.3 ± 2.9 (32)	3.5 ± 1.6 (25)
Total	4.3 ± 0.01 (51)	2.7 ± 0.01 (21)	2.6 ± 0.01 (27)

The mean contributions of carbon from C_4_ plants to TC in PM_2.5_ were 19%, 17%, 21%, and 21% at TRF, HUF, CRI, and SNU, respectively (Table 2). The largest contribution of C_4_ plants to TC in PM_2.5_ was 42% and 55% for TRF and SNU, respectively, observed on April 10, 2020. A relatively lower contribution of C_4_ plants was observed in summer (10% at TRF and 15% at SNU) compared with the other seasons (Table 2; Fig. 3b,c).

The mean contributions of carbon from C_3_ plants to TC in PM_2.5_ were 35%, 38%, 31%, and 27% at TRF, HUF, CRI, and SNU, respectively (Table 2). The highest contribution of C_3_ plants to TC in PM_2.5_ was observed in spring (49% at TRF and 32% at SNU), while in winter, the carbon contributions were 37, 34, 28, and 25% at TRF, HUF, CRI, and SNU, respectively (Table 2; Fig. 3b,c).

The concentration of TC in PM_2.5_ from each source was calculated by multiplying the concentration of TC with the individual contribution. The mean concentration of TC in PM_2.5_ derived from C_3_ plants was 2.1 μg C m^−3^ (range: 0.7–4.2 μg C m^−3^) at TRF, and 2.6 μg C m^−3^ (range 0.5–6.5 μg C m^−3^) at SNU. Although the concentration of TC in PM_2.5_ was about two-fold higher in SNU than in TRF (Table 1), the concentration of TC in PM_2.5_ derived from C_3_ plants was similar in the urban area and the pine forest (Table 2; Fig. 3b,c). In contrast, the mean concentration of TC in PM_2.5_ derived from fossil fuel was 2.1 (range: 1.1–4.2 μg C m^−3^) at TRF and 4.3 (range: 2.0–8.4 μg C m^−3^) at SNU. The concentration of TC in PM_2.5_ derived from C_4_ plants ranged from 0.2 to 2.8 with an average of 0.8 μg C m^−3^ at TRF, and ranged from 0.1 to 9.1 μg C m^−3^ with an average of 2.7 μg C m^−3^ at SNU (Table 2; Fig. 3b,c).

### Levoglucosan concentration

**Figure 4:**
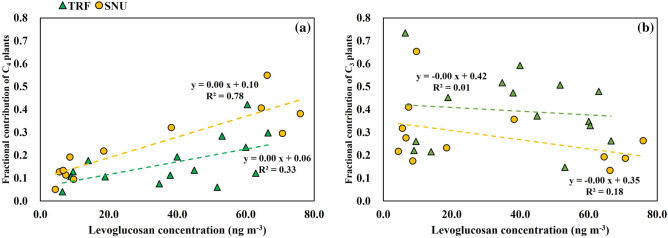
The correlation between the concentration of levoglucosan and the fractional contribution of (**a**) C_4_ plants, and (**b**) C_3_ plants.

The mean concentrations of levoglucosan were 39.8, 8.3, 34.6, and 57.5 ng m^−3^ in spring, summer, fall, and winter, respectively, at TRF (Fig. 3d), whereas those at SNU were 30.4, 6.2, 12.6, and 70.4 ng m^−3^, respectively (Fig. 3d). There was a positive correlation between the concentration of levoglucosan and fractional contribution of C_4_ plants (Fig. 4a).

## Discussion

Many studies have demonstrated that PM_2.5_ can be removed by trees^6,8–10^, which leads to urban forest expansion. However, PM_2.5_ can be indirectly produced by BVOCs emitted by plants^14,43,44^. PM_2.5_ can also be directly produced by fossil fuel or biomass burning. These contrasting views question of their relative importance in the formation of PM_2.5_. Although some studies on TC in PM_2.5_ have been conducted in East Asia, typically in urban area, they used only δ^13^C (not including Δ^14^C), different kind of dual isotopes (δ^13^C and δ^15^N), or only during a limited season not over an entire year^34,35^. In contrast, this is the first study, to our knowledge, which investigated changes of δ^13^C and Δ^14^C of PM_2.5_ over an entire year inside the forest and the urban areas in South Korea.

A review of PM_2.5_ sources based on ^14^C analysis demonstrated that the proportion of carbon sources except fossil fuels (i.e., total carbon minus carbon derived from fossil fuel) generally exceeded 30% of TC in PM collected even in urban locations^45^. If BVOCs derived from trees strongly contribute to the formation of PM_2.5_, the PM_2.5_ collected inside a forest would yield higher Δ^14^C than in urban areas. In other words, as the portion of fossil carbon increases, Δ^14^C will decrease. TC in PM_2.5_ collected in the forest (TRF) and urban areas (SNU) had depleted Δ^14^C, indicating the relative contribution of fossil carbon is constantly high (Table 2 and Fig. 2). The similar sources of PM_2.5_ in the forest and urban areas (Table 2 and Fig. 2) have been shown even during summer when the emission of BVOCs was the highest^46,47^, or during spring when the concentrations of pollen were the highest^48^ (Fig. S3 in Supplementary Information). This suggests that biogenic emission of plants could be a less important source of PM_2.5_ even during summer. The Δ^14^C values of PM_2.5_ were also similar between TRF and HUF. CRI is located next to major traffic roads (Fig. 1), where direct input of PM_2.5_ by vehicles could lower Δ^14^C. These results imply that trees can produce biogenic PM_2.5_, but most of PM_2.5_ within the forests or urban green spaces are likely to be transported from the outside.

Despite similar sources, the TC concentration in PM_2.5_ at TRF was two-fold lower than at SNU (Table 1). Considering the similar concentrations of PM_2.5_ at the monitoring sites which are located outside of forest (Fig. 3a), the lower concentrations of TC at TRF suggest that pine forests may reduce PM_2.5_ transported from the outside. The ratio of fossil carbon to the total mass of PM_2.5_ can be roughly calculated by multiplying the proportion of TC in PM_2.5_ by the proportion of fossil carbon in TC. Since ~ 30% of PM_2.5_ was carbonaceous material in general (“TC in PM_2.5_ (%)” in Table 1)^19,45^, and ~ 50% of the carbonaceous material was derived from fossil fuel (Table 2), ~ 15% of PM_2.5_ (*i.e.*, 0.3 × 0.5 = 0.15) is derived from fossil carbon. Although 15% appears small, the majority of the PM_2.5_ is still attributable to the other aerosols, such as sulfur oxides and nitrogen oxides, derived from fossil fuel burning^27,28^.

The remaining portions, ~ 50% of TC in PM_2.5_ were attributed to the non-fossil fuels. A previous study conducted at TRF during the growing season (Aug.–Oct., 2014) showed approximately 76% of TC was from non-fossil sources^35^. We further attributed the non-fossil sources of TC in PM_2.5_ to C_3_ plants- (~ 30%) and C_4_ plants-derived carbon (~ 20%) (Table 2), which was calculated over entire seasons including winter when the concentration of PM_2.5_ was the largest. The range of δ^13^C of marine aerosols could be in those between C_3_ plants and C_4_ plants^40,49^. Both C_4_ plants and marine aerosols could be selected as an end member^28,40^. However, we have selected C_4_ plants instead of marine aerosols as an endmember because of the variation of the concentration of levoglucosan (Fig. 3d). Levoglucosan is a tracer of biomass burning and thus cannot be an important component of marine aerosols^50^. The concentration of levoglucosan demonstrated clear seasonal variation, with the largest value up to 75.91 ng m^−3^ observed during winter, followed by fall and spring, and the lowest during summer (Fig. 3d). This seasonal variation has been observed in major cities in the East Asia^26,51–54^.

The concentration of levoglucosan was positively correlated with fractional contribution of C_4_ plants (up to R^2^ = 0.78, *p* < 0.05 in Fig. 4a), but not correlated with fractional contribution of C_3_ plants (Fig. 4b). This indicates that the higher concentration of levoglucosan, especially in winter was likely due to the burning of C_4_ plants (e.g., corn residues). The biomass burning of C_4_ plants has been widely considered as one of the sources of TC in PM_2.5_ because of the enriched δ^13^C and the high concentration of levoglucosan during winter^26,34,55^. The enriched Δ^14^C and δ^13^C as well as the highest concentration of levoglucosan were also reported during winter in China^26–28^.

Recent studies have demonstrated that the burning of corn residues accounted for up to 80% of the total agricultural waste burning in China^56–58^. China was the second-largest producer of corn with 23% of the global corn yield in the marketing year (Sep., 2019–Aug. 2020), following the U.S. very closely (https://www.fas.usda.gov/data/world-agricultural-production). In contrast, corn production in South Korea is substantially lower than in China. It has been also reported that the burning of corn residues was ~ 10% of the total agricultural waste burning in several rural areas of South Korea^59^, although agricultural waste burning over the entire South Korea is rarely reported. Since northwesterly winds around Korea are predominant during winter as the Siberian High (i.e., Siberian Anticyclone) develops (^60^; Fig. S5 in Supplementary Information), TC derived from C_4_ biomass burning could be not only generated within South Korea but also transported from North Korea and/or China^61,62^.

Su et al.^63^ quantified the proportion of nine sources of PM_2.5_ by using a multivariate factor analysis in the western urban area of Shenzhen, China in the fall (Sep.– Nov., 2019). Among them, the contribution of biomass burning to the total concentration of PM_2.5_ was only 11%^61^. The results of dual carbon isotope and levoglucosan analyses in this study showed about 20% of the carbonaceous component of PM_2.5_ might be derived from the burning of C_4_ plants. Considering TC accounted for ~ 30% of PM_2.5_ [Table 1^19,45^, the carbon derived from biomass burning of C_4_ plants could contribute at least ~ 6% (*i.e.*, 0.2 (above 20%) × 0.3 (above 30%) = 0.06) of the total PM_2.5_, while inorganic materials can be attributed to another fraction of PM_2.5_^64^.

The carbon derived from C_3_ plants was estimated at only ~ 9% of PM_2.5_ in TRF and SNU, which can be attributed to biogenic emissions by trees or biomass burning of C_3_ plants. The concentrations of TC derived from C_3_ plants were similar at TRF and SNU, whereas the concentrations of TC derived from fossil-carbon and C_4_ plants in the urban areas were about two-fold the levels at TRF (Table 2; Fig. 3b,c). Thus, biogenic emission of pines is not likely to be an important source of PM_2.5_ in the forest, suggesting that trees act as a bio-filter of PM_2.5_ in urban areas.

## Conclusions

We analyzed dual carbon isotope ratios and levoglucosan concentrations in a pine forest and in urban areas to provide source apportionment of TC in PM_2.5_. The δ^13^C and Δ^14^C of TC in PM_2.5_ indicated similar sources of carbon in PM_2.5_ in the forest and urban areas, although the total concentration of TC in PM_2.5_ was approximately two-fold lower in the forest than in urban areas. TC in PM_2.5_ was predominantly affected by human activities such as fossil-fuel combustions and C_4_ biomass burning rather than emissions from C_3_ plants. These results suggest that strategies to reduce atmospheric PM_2.5_ should focus on fossil fuel combustion and biomass burning instead of biogenic emissions from trees.

## Supplementary Information

Supplementary Figures.

## Data Availability

The data used to support the findings of this study are included within the article and its supplementary information files.
