# Supplementary material for: Fossil and non-fossil sources of the carbonaceous component of PM2.5 in forest and urban areas
Source: Sci Rep. 2023 Apr 4;13:5486. doi: 10.1038/s41598-023-32721-2 (PMC10073123; doi:10.1038/s41598-023-32721-2)
Supplement: Supplementary file 1 — Supplementary Figures. [file 41598_2023_32721_MOESM1_ESM.docx]

***Supplementary Information***

Fossil and non-fossil sources of the carbonaceous component of PM_2.5_ in forest and urban areas

Ji-Yeon Cha^a^, Kyuyeon Lee^a^, Seung-Cheol Lee^a^, Eun-Ju Lee^b^, Kwang-Jin Yim^c^, Ilhan Ryoo^d^, Minhye Kim^d^, Jinho Ahn^c^, Seung-Muk Yi^d, e^, Chan-Ryul Park^f^ and Neung-Hwan Oh^a, b*^

^a^ Department of Environmental Planning, Graduate School of Environmental Studies, Seoul National University, Seoul 08826, Republic of Korea

^b^ Environmental Planning Institute, Seoul National University, Seoul 08826, Republic of Korea

^c^ School of Earth and Environmental Sciences, Seoul National University, Seoul 08826, Republic of Korea

^d^ Department of Environmental Health, Graduate School of Public Health, Seoul National University, Seoul 08826, Republic of Korea

^e^ Institute of Health and Environment, Seoul National University, Seoul 08826, Republic of Korea

^f^ Urban Forests Division, National Institute of Forest Science, Seoul 02455, Republic of Korea

* Corresponding author. E-mail address: [onh@snu.ac.kr](mailto:onh@snu.ac.kr) (N.-H. Oh).

**1. MCMC-based Bayesian model**

In this model, prior information was derived from the IsoSource model (Fig. S1). Without the prior information, the fractional contribution of fossil fuel source in the Bayesian model was inconsistent with the results of the radiocarbon analysis (Fig. S2). We used prior information to improve model estimates.


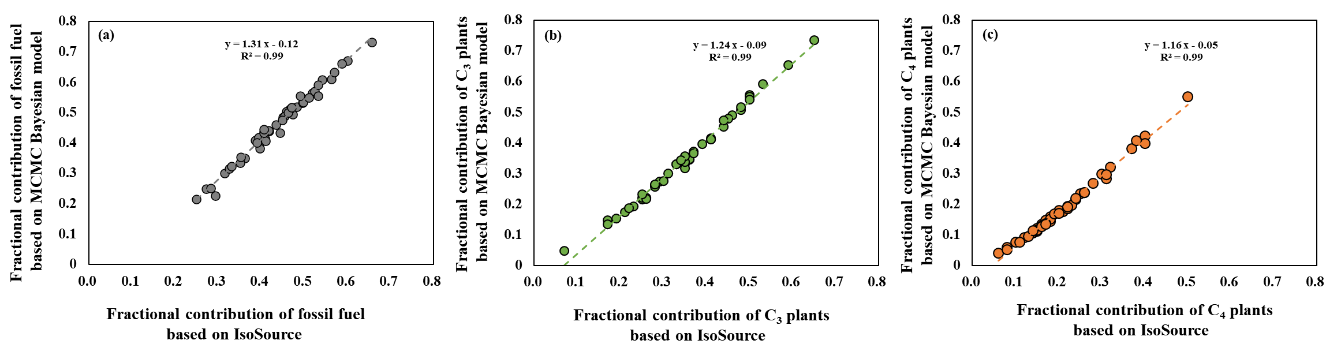


**Figure S1.** Fractional contribution of (a) fossil fuels, (b) C_3_ plants, and (c) C_4_ plants based on IsoSource model and MCMC Bayesian model with prior information.


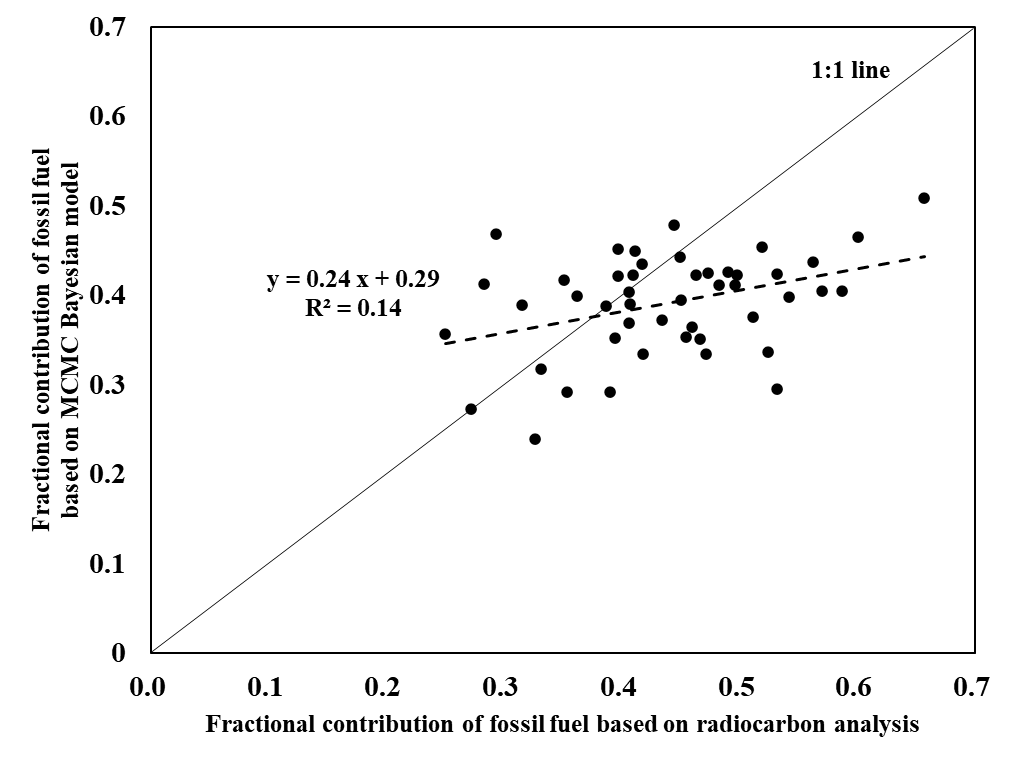


**Figure S2.** The fractional contribution of fossil fuel sources based on radiocarbon analysis and MCMC Bayesian model without prior information.

**2. PM_2.5_ sample with high Δ^14^C**

The high Δ^14^C, 589.7‰, for PM_2.5_ collected on January 14, 2019 at SNU was also observed when PM_2.5_ sample was independently collected on the same date at Korea University (KU) (16 km away from SNU) Seoul, South Korea by another researcher (personal communication). The radiocarbon analysis of the KU sample was conducted at National Electrostatics Corporation (Middleton, WI, USA), whereas our SNU sample was analyzed at National Ocean Sciences Accelerated Mass Spectrometry (Woods Hole, MA, USA).

In Wilmington, USA, the Δ^14^C of PM_2.5_ was up to 2,475‰ during December in 2007 and in Bondville, Illinois, USA, the Δ^14^C was up to 1,482‰ during summer in 2004 [1]. Such Δ^14^C values are not common, but were reported in PM and precipitation samples in some regions [2]. Although the radiocarbon analysis is a useful tool for source apportionment, caution is needed when interpreting the sources of carbonaceous component in PM_2.5_. Our analysis does not include the high Δ^14^C sample (589.7‰).

**3. Temporal variations of dual carbon isotope ratios of PM_2.5_**


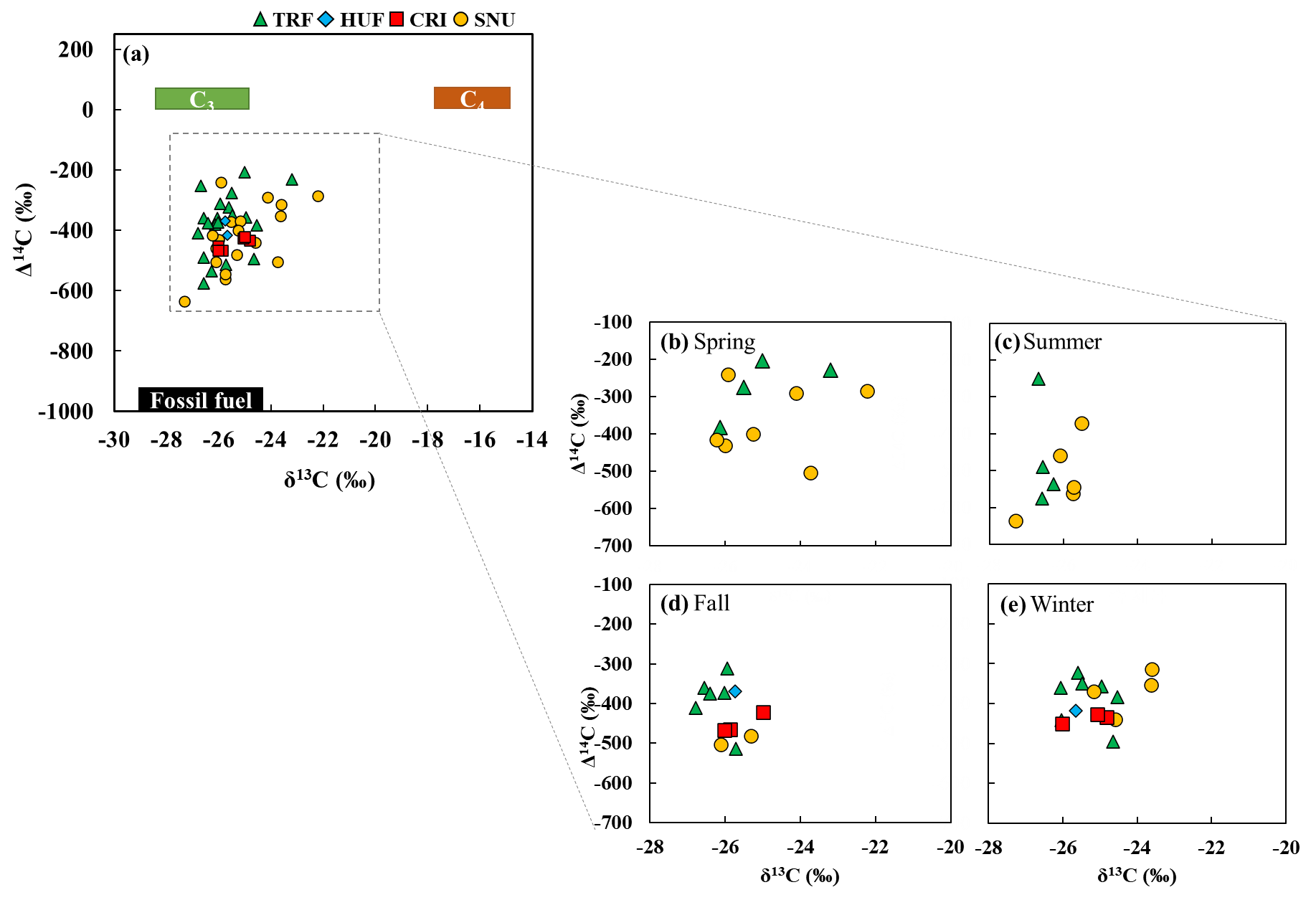


**Figure S3.** (a) Isotopic source diagram for the PM_2.5_ samples. The black, green, and brown bars indicate three endmembers including fossil-, C_3_ plants-, and C_4_ plants-derived carbon, respectively. The seasonal variations of δ^13^C and Δ^14^C values of PM_2.5_ are presented in (b–d).


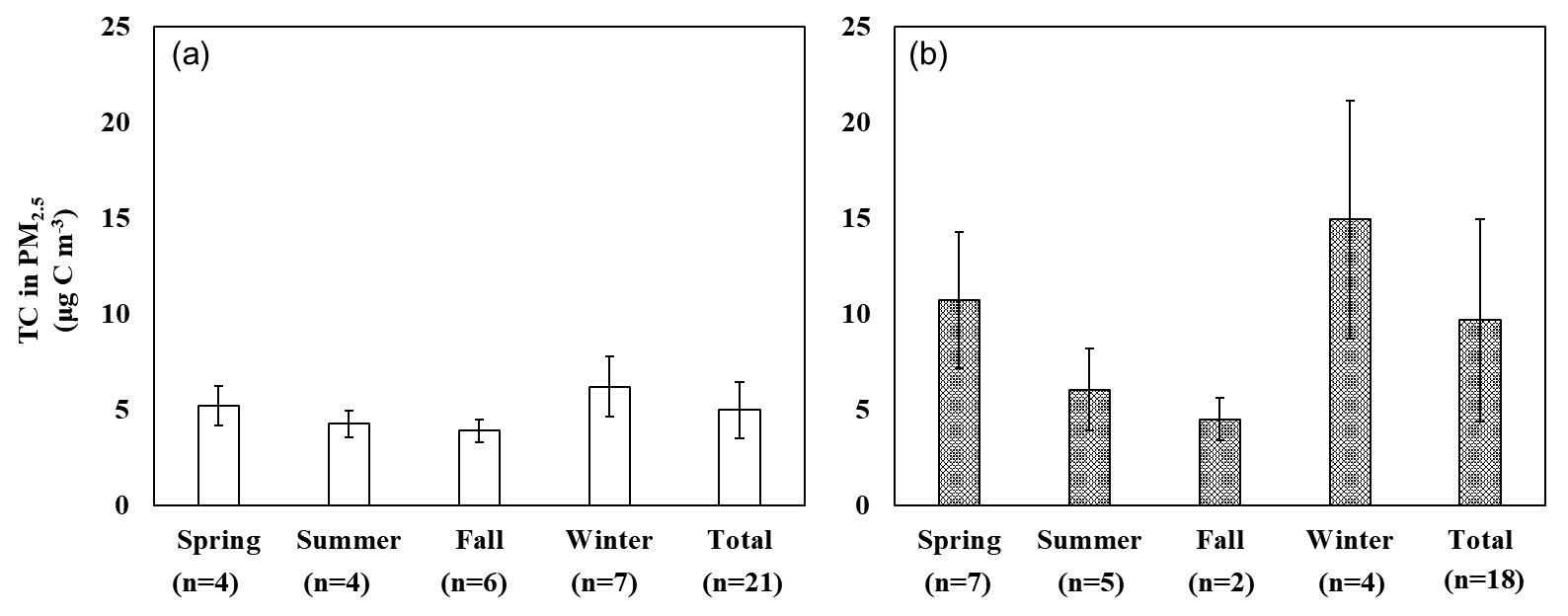


**Figure S4.** Seasonal concentrations (μg C m^-3^) of TC in PM_2.5_ at (a) TRF and (b) SNU. The vertical lines represent the standard deviation.


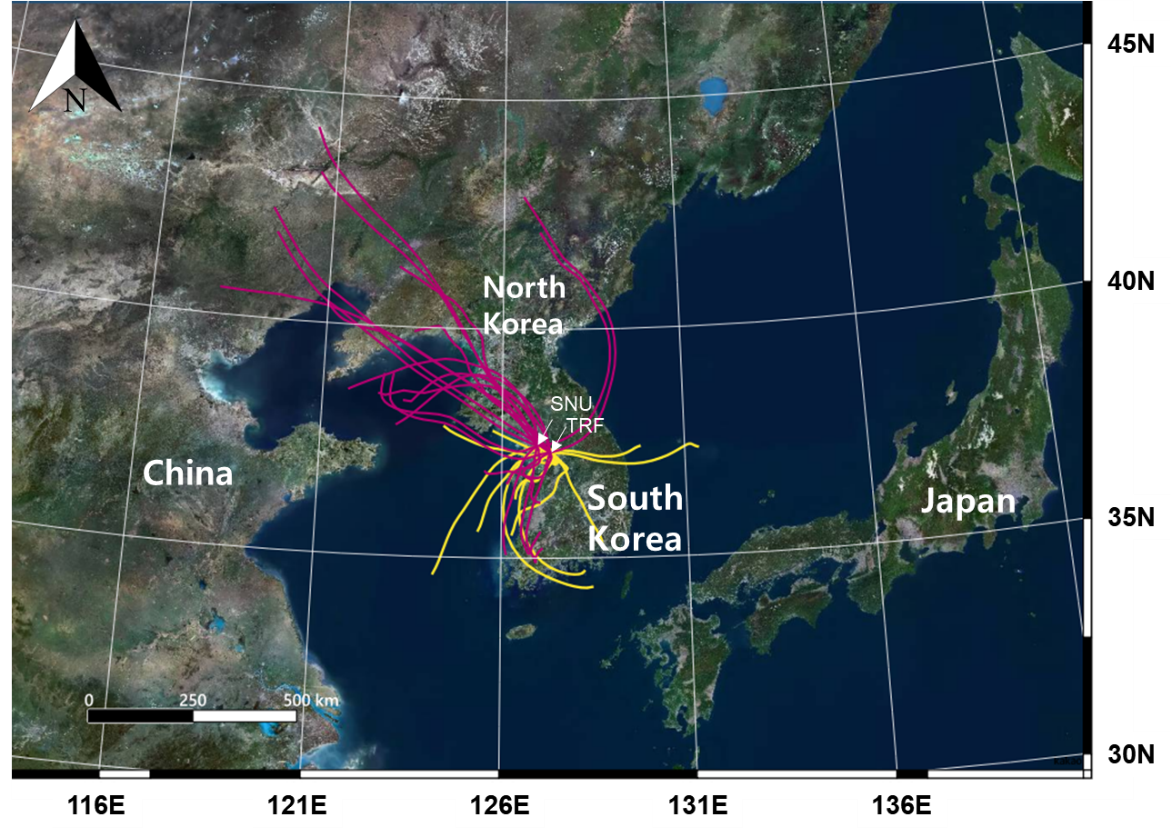


**Figure S5.** The 24-h backward trajectories of air mass reaching TRF and SNU at a height of 500 meters above ground level. The concentrations of levoglucosan were higher than 30 ng m^-3^ in spring and winter (pink lines) while in the other seasons were below this level (yellow lines). Shown trajectories were produced using the Hybrid Single Particle Lagrangian Integrated Trajectory (HYSPLIT ver 5.2.1, http://ready.arl.noaa.gov/HYSPLIT.php) model developed at the Air Resources Laboratory of NOAA [3] and the global data assimilation system (GFSQ, ftp://arlftp.arlhq.noaa.gov/pub/archives/gfs0p25/) database. The trajectory map was generated by QGIS 3.28.1 (https://qgis.org/ko/site/).

**References**

1. Buchholz, B. A., Fallon, S. J., Zermeño, P., Bench, G., & Schichtel, B. A. Anomalous elevated radiocarbon measurements of PM_2.5_.  *Nucl. Instrum. Methods Phys. Res. B*. **294**, 631-635 (2013).
2. Cha, J. Y. et al. High dissolved organic radiocarbon in precipitation during winter and its implication on the carbon cycle. *Sci. Total. Environ.* **742**, 140246; 10.1016/j.scitotenv.2020.140246 (2020).
3. Draxler, R.R. & Hess, G. An overview of the HYSPLIT_4 modelling systemfor trajectories. *Aust. Meteorol. Mag.* **47(4)**, 295–308 (1998).
